# α-TEA-induced death receptor dependent apoptosis involves activation of acid sphingomyelinase and elevated ceramide-enriched cell surface membranes

**DOI:** 10.1186/1475-2867-10-40

**Published:** 2010-10-25

**Authors:** Jing Li, Weiping Yu, Richa Tiwary, Sook-Kyung Park, Ailian Xiong, Bob G Sanders, Kimberly Kline

**Affiliations:** 1 School of Biological Sciences/C0900, University of Texas at Austin, Austin, TX 78712, USA; 2 Department of Nutritional Sciences/A2703, University of Texas at Austin, Austin, TX 78712, USA

## Abstract

**Background:**

Alpha-tocopherol ether-linked acetic acid (α-TEA), an analog of vitamin E (RRR-alpha-tocopherol), is a potent and selective apoptosis-inducing agent for human cancer cells *in vivo *and *in vitro*. α-TEA induces apoptosis via activation of extrinsic death receptors Fas (CD95) and DR5, JNK/p73/Noxa pathways, and suppression of anti-apoptotic mediators Akt, ERK, c-FLIP and survivin in breast, ovarian and prostate cancer cells.

**Results:**

In this study, we demonstrate that α-TEA induces the accumulation of cell surface membrane ceramide, leading to co-localization with Fas, DR5, and FADD, followed by activation of caspases-8 and -9 and apoptosis in human MDA-MB-231 breast cancer cells. α-TEA treatment leads to increased acid sphingomyelinase (ASMase) activity by 30 min, peaking at 4 hrs, which is correlated with ASMase translocation from cytosol to the cell surface membrane. Functional knockdown of ASMase with either the chemical inhibitor, desipramine, or siRNA markedly reduces α-TEA-induced cell surface membrane accumulation of ceramide and its co-localization with Fas, DR5, and FADD, cleavage of caspases-8 and -9 and apoptosis, suggesting an early and critical role for ASMase in α-TEA-induced apoptosis. Consistent with cell culture data, immunohistochemical analyses of tumor tissues taken from α-TEA treated nude mice bearing MDA-MB-231 xenografts show increased levels of cell surface membrane ceramide in comparison to tumor tissues from control animals.

**Conclusion:**

Taken together, these studies demonstrate that ASMase activation and membrane ceramide accumulation are early events contributing to α-TEA-induced apoptosis *in vitro *and perhaps *in vivo*.

## Background

Specialized domains of cell surface membranes termed lipid rafts/platforms or lipid microdomains play a key role in the spatial organization of receptors and signaling molecules [1-3]. Ceramide-enriched lipid microdomains can be formed by increased amounts of ceramide in cell surface membranes which is regulated by sphingomyelinase (SMase) hydrolysis of raft-associated sphingomyelin to ceramide [2,3]. Once ceramide-enriched microdomains are formed, Fas/FasL and DR5/TRAIL as well as other signaling mediators become clustered and can be activated in either a ligand-dependent or -independent manner [4-6]. Clustering of Fas or DR5 can result in rapid association of these receptors with adapter protein FADD [7], which recruits the initiation caspase-8 to its death effector domain, initiating apoptosis via death inducing signaling complex (DISC) [8]. Prevention of raft reorganization into larger platforms by inactivation of acid sphingomyelinase (ASMase), renders cells resistant to Fas clustering and apoptosis [9], indicating that ASMase/ceramide plays an important role in death receptor-mediated apoptosis.

α-TEA (RRR-alpha-tocopherol ether-linked acetic acid analog), a derivative of vitamin E (RRR-alpha-tocopherol), is a potent pro-apoptotic agent for a variety of human cancer cells, including breast cancer cells *in vivo *and *in vitro *[10-24]. α-TEA does not induce apoptosis in normal human mammary epithelial cells (HMEC) or immortalized, nontumorigenic breast cells (MCF-10A cells) [17] and produces no signs of toxicity when given to mice [10,11,19,24,25]. Previous studies utilizing various functional knockdown strategies (siRNA, neutralizing antibodies, transfection of dominant negative proteins and small chemical inhibitors) show that α-TEA-induced apoptosis in human breast, ovarian and prostate cancer cells is mediated by Fas and DR5 death receptor signal transduction via FADD or Daxx and involves both caspase-8 and mitochondrial-dependent signal transduction events [15,16,18,20,21]. However, mechanisms involved in α-TEA induced Fas- and DR5-dependent apoptotic signaling are not fully understood. Here we report that α-TEA initiates activation of ASMase via translocation from the cytosol to the cell surface membrane, leading to accumulation and aggregation of membrane ceramide domains enriched with death signaling mediators; Fas, DR5 and their adaptor FADD, as an essential early event in α-TEA induced Fas and DR5 dependent apoptosis.

## Materials and methods

### Chemicals

α-TEA (F.W. = 488.8; 40 μM) was dissolved in ethanol as stock solution as described previously [10]. Desipramine, an ASMase inhibitor, GW4869, a neutral SMase (NSMase) inhibitor, and fumonisin B1, a ceramide synthase inhibitor, were obtained from Sigma-Aldrich (St. Louis, MO). Myriocin, a serine palmitoyltransferase inhibitor of the de novo ceramide synthesis pathway, was obtained from BioMol (Plymouth Meeting, PA).

### Cell Culture

Triple-negative (ER^-^, PR^-^, Her-2^-^) MDA-MB-231 human invasive ductal carcinoma, basal-like breast cancer cells (American Type Culture Collection, Manassas, VA) were cultured in MEM media with 10% FBS. For experiments, FBS was reduced to 2%. All cells were plated at 1.5 × 10^5 ^cells/well in 12 well plates for apoptosis analyses, 3 × 10^5 ^cells/well in 6 well plates for immunofluorescence staining and FACS analyses, and 2 × 10^6 ^cells/100 mm^2 ^or 6 × 10^6 ^cells/150 mm^2 ^dishes for western blot analyses. Cells were allowed to attach overnight before treatment initiation. The cells were treated with α-TEA at 40 μM with equivalent level of solvent (0.1% ethanol) serving as vehicle control (VEH).

### Quantification of apoptosis

Apoptosis was quantified by Annexin V-FITC/PI assay (Invitrogen, Carlsbad, CA) following manufacturer's instructions. The Annexin V-FITC/PI assay measures amount of phosphatidylserine on the outer surface of the plasma membrane (a biochemical alteration unique to membranes of apoptotic cells) and amount of propidium iodide (PI), a DNA binding dye that does not cross the plasma membrane of viable cells but readily enters dead cells or cells in the late stages of apoptosis. Fluorescence was measured using Fluorescence Activated Cell Sorter (FACS) analyses with a FACSCalibur flow cytometer. Data were analyzed using CellQuest software (BD Biosciences, San Jose, CA). Cells displaying phosphatidylserine on their surface (positive for Annexin-V fluorescence) were considered to be apoptotic.

### Small interfering RNA (siRNA) transfection

A scrambled RNA duplex that does not target any known genes was used as the non-specific negative control for RNAi (referred to as control siRNA). Transfection of MDA-MB-231 cells with siRNAs to ASMase or control (Ambion, Austin, TX) was performed in 100 mm^2 ^cell culture dishes at a density of 2 × 10^6 ^cells/dish using Lipofectamine-2000 (Invitrogen) and siRNA duplex, resulting in a final siRNA concentration of 30 nM following the company's instructions. One day after transfection initiation, the cells were re-cultured in 100 mm^2 ^dishes at 2 × 10^6 ^cells/dish and incubated for 1 day followed by treatments.

### Western Blot analyses

Whole cell protein extracts were prepared and examined by western blotting as described previously [26]. Proteins at 30-50 μg/lane were separated by SDS-PAGE and transferred to nitrocellulose (Optitran BA-S supported nitrocellulose, Schleicher and Schuell, Keene, NH). Antibodies (1 μg/ml) to the following proteins were used: poly (ADP-ribose) polymerase (PARP) (Santa Cruz Biotechnology, Santa Cruz, CA), caspase-8 and caspase-9 (Cell Signaling Technology, Beverly, MA), and glyceraldehyde-3-phosphate dehydrogenase (GAPDH; made in house). Horseradish peroxidase conjugated goat-anti-rabbbit or goat-anti-rabbbit secondary antibodies (1:2000 dilution) (Jackson ImmunoResearch Laboratories, Rockford, IL) was used.

### Immunofluorescent staining detection of ASMase, ceramide, DR5, Fas and FADD

Cellular location of ASMase, ceramide, DR5, Fas and FADD was determined using immunofluorescent staining. Treated and control cells were washed two times with PBS and fixed with fresh 4% paraformaldehyde in PBS for 15 min at room temperature. After 3 washes with PBS, cells were permeated with 0.1% Triton X-100 for 15 min at room temperature followed by incubating the cells with blocking buffer (2% FBS in PBS) for 1 hr, and then incubating the cells with reaction buffer (1% FBS in PBS) overnight at 4°C with antibodies (20 μg/ml) to DR5 (Abcam Inc, Cambridge, UK), FADD (BD Biosciences, San Jose, CA), ASMase and Fas (Santa Cruz Biotechnology), and antibody (4 μg/ml) to ceramide (Sigma-Aldrich). Samples were washed three times with PBS and stained for 1 hr with Texas Red donkey anti-rabbit IgG (Santa Cruz Biotechnology) for DR5, Alexa Fluor 594 goat anti-mouse IgG (Invitrogen) for Fas and FADD, Alexa Fluor 488 goat anti-mouse IgM and FITC-conjugated rabbit anti-goat IgG (Jackson ImmunoResearch Laboratories) for ceramide and ASMase at 10 μg/ml, respectively, in reaction buffer. Cells were washed three times with PBS. Images were acquired using fluorescent microscopy or laser-scanning confocal microscope.

### Detection of ceramide expression in cell surface membranes of living cells

The expression of cell surface membrane ceramide was determined by immunostaining analyses using FACS or fluorescent microscopy. 2 × 10^6 ^cells were collected, washed in ice-cold PBS, and blocked with blocking buffer (2% FBS in PBS) for 20 min on ice. Cells were re-washed and incubated for 1 hr with 4 μg/ml monoclonal ceramide antibody (15B4, Sigma) on ice, followed by ice-cold PBS washing and incubation with Alexa fluor 488 goat anti-mouse IgM at 10 μg/ml on ice for 30 min. After two PBS washes, cells were analyzed immediately using a FACScan flow cytometer or by immunofluorescence microscopy for cell surface membrane ceramide.

### ASMase Activity Assay

ASMase activity was determined using an acid sphingomyelinase assay kit (Echelon Biosciences Inc. Salt Lake City, UT) following company's instructions. Cells were disrupted in ice-cold lysis buffer [150 mM NaCl, 50 mM Tris pH 7.4, 0.6% Triton X-100, and protease inhibitor cocktail (Sigma-Aldrich)] for 20 min. Cellular debris was removed after centrifugation at 10,000 × g for 5 min, and 20 μg protein was used to determine ASMase activity. Reaction was stopped after incubation at 37°C for 2 hrs and analyzed using a fluorescence plate reader at 260 nm excitation and 460 nm emission.

### Immunohistochemical detection of membrane ceramide

Archived formalin fixed tumor tissue from a MDA-MB-231 xenograft study [27] was used to determine if α-TEA induces elevated membrane ceramide *in vivo*. Mice were euthanized after 24 days of α-TEA dietary treatment (378 mg α-TEA/kg diet). Immediately upon collection, tumors were fixed in formalin and archived for immunohistochemical analyses [27]. Deparaffinized 5 micron sections were examined by IHC employing a monoclonal antibody against ceramide (MID 15B4; Sigma-Aldrich). Three tumors from control and α-TEA treatment groups were analyzed. Deparaffinized sections were placed in 10 mM sodium citrate buffer (pH 6.0) and boiled 10 min for antigen unmasking. After rinsing, sections were placed in PBS followed by incubating with blocking buffer (2% FBS, 0.3% Triton X-100 in PBS) for 1 hr. After removal of blocking buffer, cells were incubated with primary mouse anti-ceramide antibody (4 μg/ml in 1% FBS/PBS) or mouse IgM (10 μg/ml for negative control) overnight at 4°C. After 3 washes with PBS for 10 min each, the sections were stained with DakoCytomation LSAB + System-AP following the company's instruction (Dako Carpinteria, CA) and DAPI.

### Statistical Analysis

The student's *t*-test was used to determine statistical differences between treatment and control values. Differences were considered statistically significant at *p *< 0.05.

## Results

### α-TEA induces apoptosis in MDA-MB-231 human breast cancer cells

**Figure 1:**
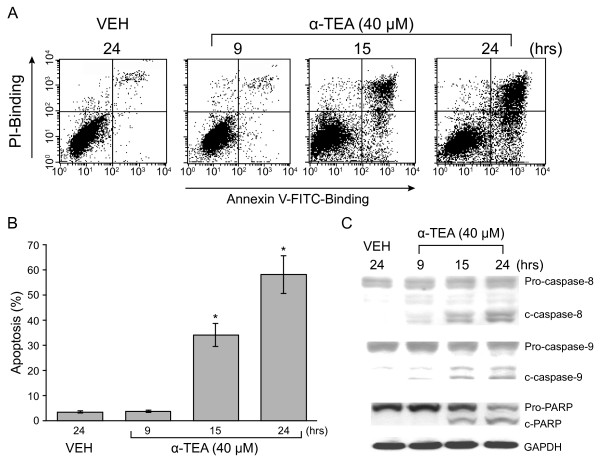
**α-TEA induces apoptosis in human breast cancer cells**. A & B, MDA-MB-231 cells were treated with 40 μM α-TEA for 9, 15, and 24 hrs. Apoptosis was determined by FACS/Annexin V assay (A) and depicted in graphic form (B). C, Western blot analyses were used to detect cleavage of caspases-8, -9 and PARP. Data in A & C are representative of three experiments. Data in B is presented as means ± S.D. of three independent experiments.

α-TEA induces MDA-MB-231 cells to undergo apoptosis in a time dependent manner as measured by FACS/Annexin V/PI analyses. Apoptosis was detected at 15 hrs after treatment with 40 μM α-TEA (Figure 1A &1B). α-TEA treatment for 9 hrs did not show a significant increase in percentage of apoptotic cells in comparison with VEH control, whereas α-TEA treatments for 15 and 24 hrs produced a high percentage of apoptotic cells, approximately, 33% and 57%, respectively, suggesting that apoptosis occurred between 9-15 hrs. Data from western immunoblotting analyses show cleavage of caspases-8 and -9 in lower levels at 9 hrs, and higher levels at 15 and 24 hrs after treatment (Figure 1C). Cleavage of PARP, an apoptotic marker, was detected at 15 and 24 hrs after treatment (Figure 1C).

### α-TEA induces increased level of ceramide in cell surface membrane

**Figure 2:**
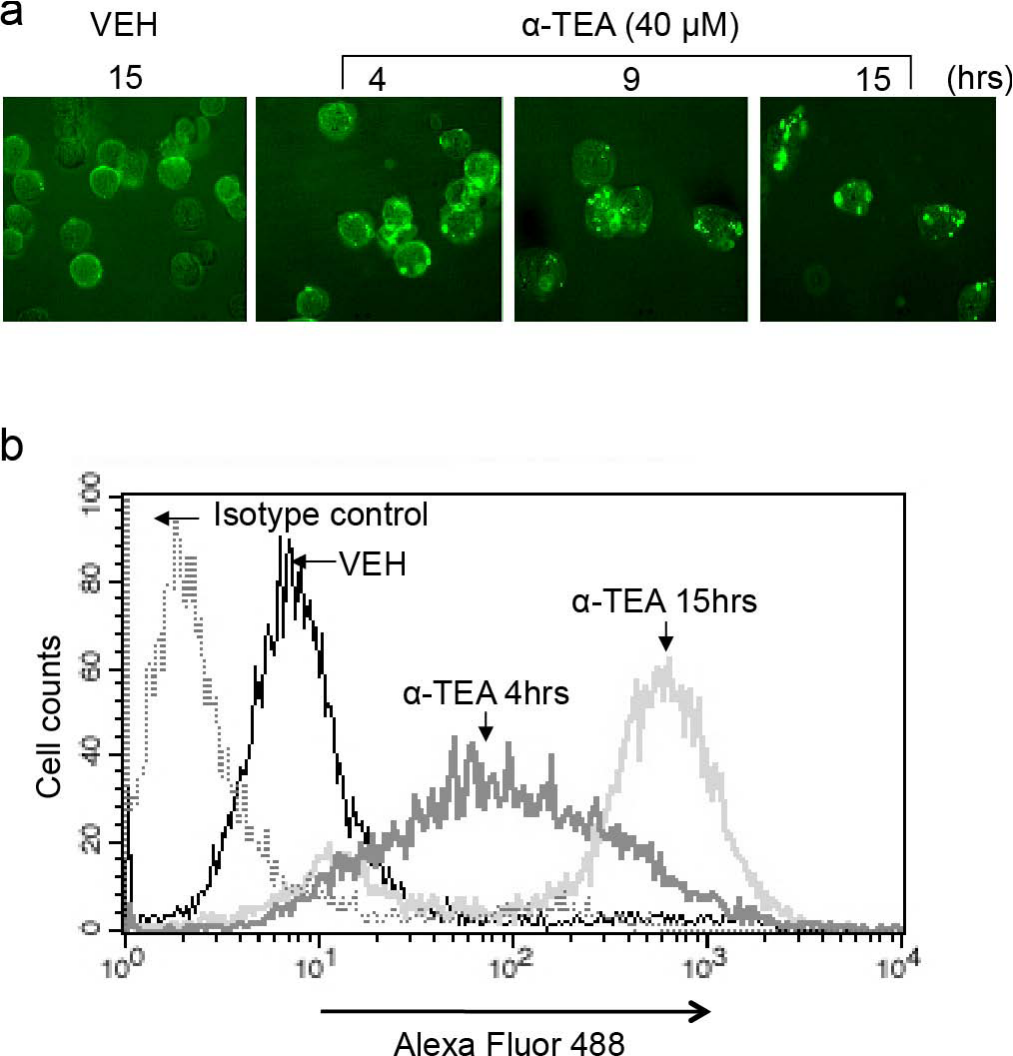
**α-TEA induced increased levels and aggregation of ceramide in cell surface membranes**. A, MDA-MB-231 cells were treated with 40 μM α-TEA for 4, 9 and 15 hrs (15 hrs for vehicle control). The living cells were collected and immunostained with antibody to ceramide. Cellular membrane ceramide was assessed by fluorescence microscope. B, Samples from Figure 2a at 4 and 15 hrs time points were also assessed by FACS for membrane expression of ceramide. All images are representative of three independent experiments.

To see if α-TEA induces an increased level of ceramide in the cell surface membrane, membrane ceramide expression in α-TEA treated MDA-MB-231 cells was determined by fluorescent microscopy or FACS analyses of cells stained for ceramide (Figure 2). Microscopic analyses showed that ceramide was aggregated in cell surface membranes starting as early as 4 hrs and continued to be elevated at 15 hrs after α-TEA treatment in comparison with VEH control (Figure 2A). In agreement with the microscopic morphological data, FACS analyses showed that cell surface membrane level of ceramide in α-TEA treated cells were increased at 4 and 15 hrs (Figure 2B). These data show that α-TEA induces increased and prolonged levels of ceramide, which is aggregated in the cell surface membrane as early as 4 hrs.

### α-TEA induces accumulation of DR5, Fas and FADD in elevated ceramide-enriched membrane

**Figure 3:**
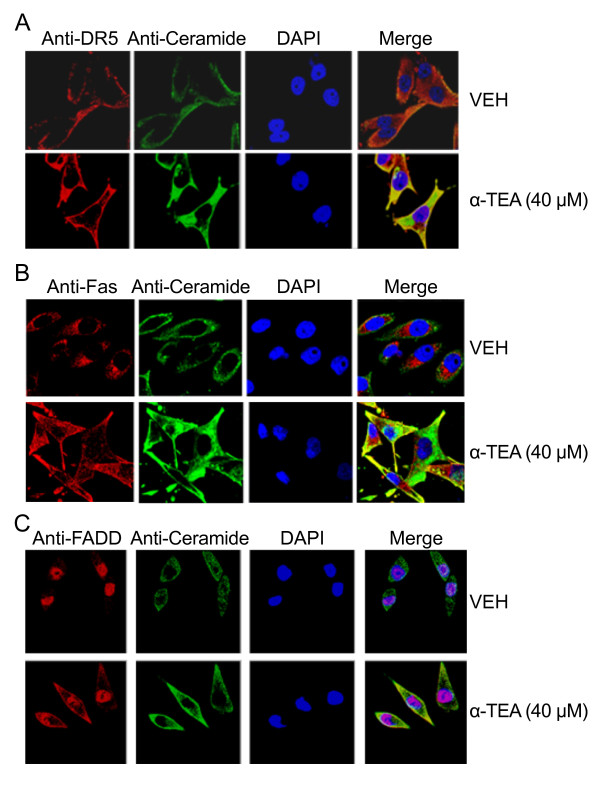
**α-TEA induces accumulation of DR5, Fas, FADD and ceramide in cell surface membranes**. A, B & C, Cells were treated with 40 μM α-TEA or vehicle (VEH) for 15 hrs and cellular localization of DR5, Fas, FADD (red channel) and ceramide (green channel) were detected by immunostaining in paraformaldehyde fixed cells. Merge = overlay of ceramide (green channel) + DAPI (blue channel) + DR5 or Fas, or FADD (red channel). All data are representative images of three separate experiments.

The effects of α-TEA on the expression of cell membrane death receptors Fas, DR5 and death adaptor protein FADD as well as membrane ceramide were examined using immunofluorescent staining procedures (Figure 3A, B, &3C). α-TEA induced increased levels of ceramide in cell surface membrane in comparison to VEH control and overlay (merge) of ceramide and DAPI-stained nuclei with DR5, Fas, or FADD showed the death mediators to be co-localized with ceramide (Figure 3A, B, &3C). These data demonstrate that α-TEA induced accumulation of DR5, Fas and FADD in ceramide enriched cell surface membrane, suggesting ceramide may be playing an important role in α-TEA-induced apoptosis.

### α-TEA activation of ASMase is an early event

**Figure 4:**
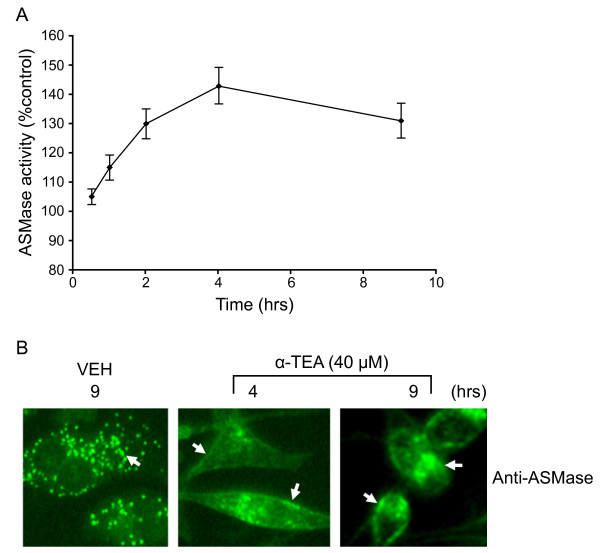
**α-TEA induces ASMase activation**. A, ASMase activity was detected using the Acid Sphingomyelinase Assay kit (Echelon) at different time points following treatment with 40 μM α-TEA. B, Cellular localization of ASMase following treatment with 40 μM α-TEA for 4 and 9 hrs (9 hrs vehicle treatment) was detected (green channel) using immunofluorescent staining of paraformaldehyde fixed cells. Arrows in α-TEA treated cells point to membrane ASMase versus arrows in vehicle control point to cellular dispersion of ASMase.

To determine if ASMase is activated by α-TEA, ASMase activity was measured using a colorimetric sphingomyelinase assay kit. ASMase activity was increased from 105% at 30 min to a peak of 143% at 4 hrs and continued to be elevated at 9 hrs after α-TEA treatment in comparison to VEH control (control = 100%) (Figure 4A). It is well established that activation of ASMase is coupled with its translocation from intracellular compartments to the cell surface membrane [2]; therefore, we determined whether activity of ASMase was associated with relocalization of ASMase from the cytosol to the membrane. Confocal microscopic imaging revealed that α-TEA induces ASMase relocation from punctuate cytosol location (see in VEH treated cells) to a diffuse cell surface membrane location (arrows) by 4 hrs with diffuse membrane location remaining at 9 hrs following treatment (Figure 4B). These data demonstrate that α-TEA activation of ASMase via translocation of it from cytosol to membrane is an early event.

### ASMase inhibitor desipramine blocks α-TEA-induced apoptosis

**Figure 5:**
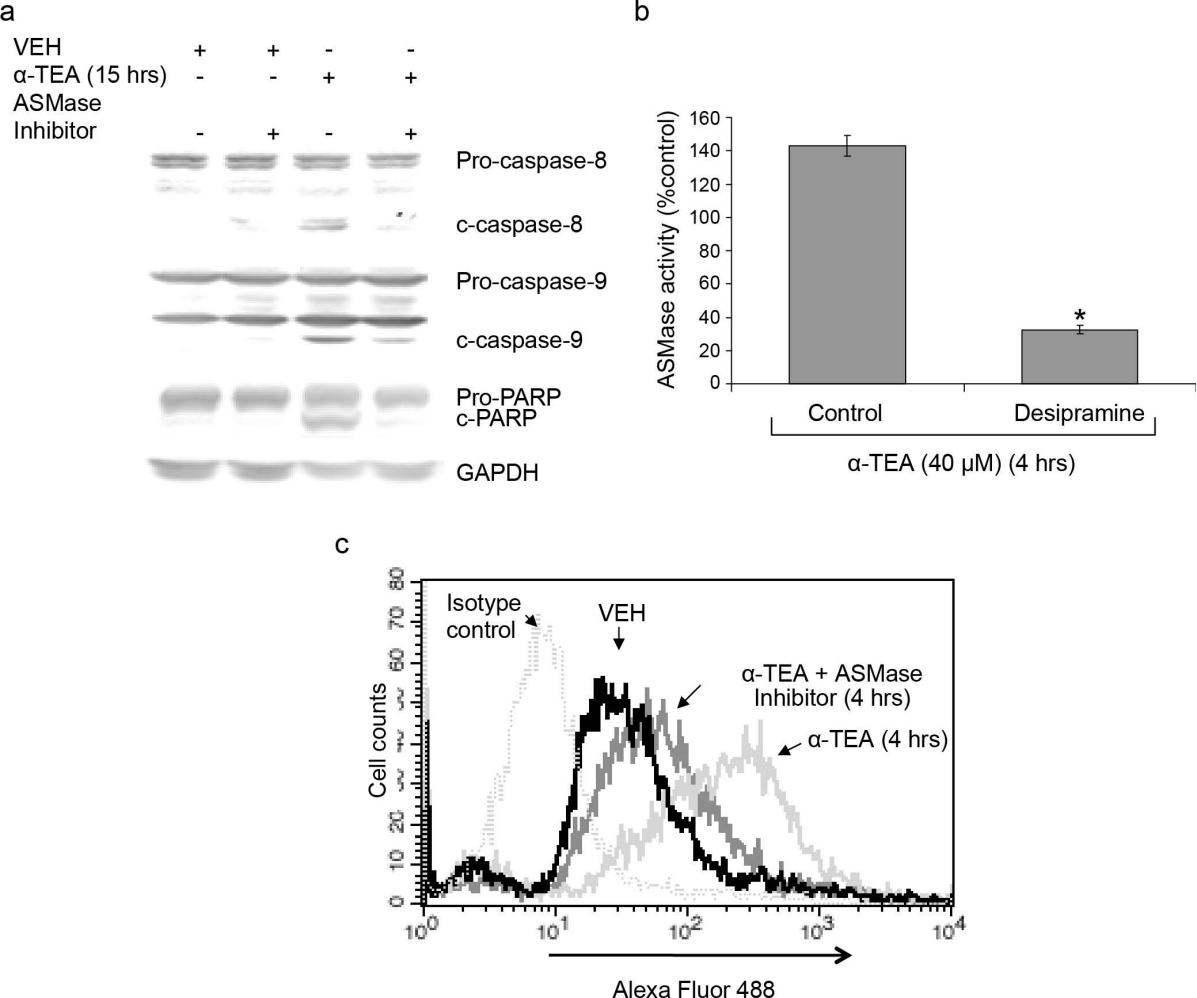
**ASMase inhibitor desipramine blocks α-TEA-induced apoptosis**. MDA-MB-231 cells were pre-treated with 12.5 μM desipramine for 2 hrs followed by treating the cells with 40 μM α-TEA for different time periods. A, Western blot analyses of cellular lysates were performed to detect cleavage of caspases-8 &-9 and PARP. B, ASMase activity was detected using the Acid Sphingomyelinase Assay kit (Echelon). C, Ceramide levels in cell surface membranes were detected by FACS analyses after reaction of living cells with primary ceramide antibody and fluorescence labeled secondary antibody after 4 hours of α-TEA treatment. Data are representative of three independent experiments. Data in **B **are presented as means ± S.D. of three independent experiments. (* = Significantly different, *P *< 0.05).

To investigate whether ASMase is involved in α-TEA-induced apoptosis and if it produces increased levels of ceramide at the cell surface, the sphingomyelin hydrolysis pathway was blocked by ASMase inhibitor desipramine. Inhibition of ASMase blocked α-TEA-induced apoptosis as documented by reduced ability of α-TEA to induce cleavage of caspases 8 & 9 and PARP (Figure 5A). In contrast GW4869, an inhibitor of sphingomyelin hydrolysis, myriocin, an inhibitor of de novo ceramide synthesis and fumonisin B1, an inhibitor of the ceramide salvage pathway had no effect on α-TEA-induced apoptosis (data not shown). Assessment of ASMase activity shows that desipramine blocks α-TEA-induced increases in ASMase activity by 77% (Figure 5B), confirming that desipramine is an effective ASMase inhibitor. FACS analyses of ceramide stained cells show that desipramine blocks the increase of ceramide at the cell surface membrane in comparison with α-TEA treated cells in the absence of ASMase inhibitor (Figure 5C), indicating that anti-ceramide antibody (15B4) is specific for recognizing membrane ceramide. Taken together, these data suggest that ASMase is involved in α-TEA-induced apoptosis and is involved in α-TEA-mediated increases of ceramide at the cell surface.

### siRNA to ASMase blocked α-TEA-induced apoptosis

**Figure 6:**
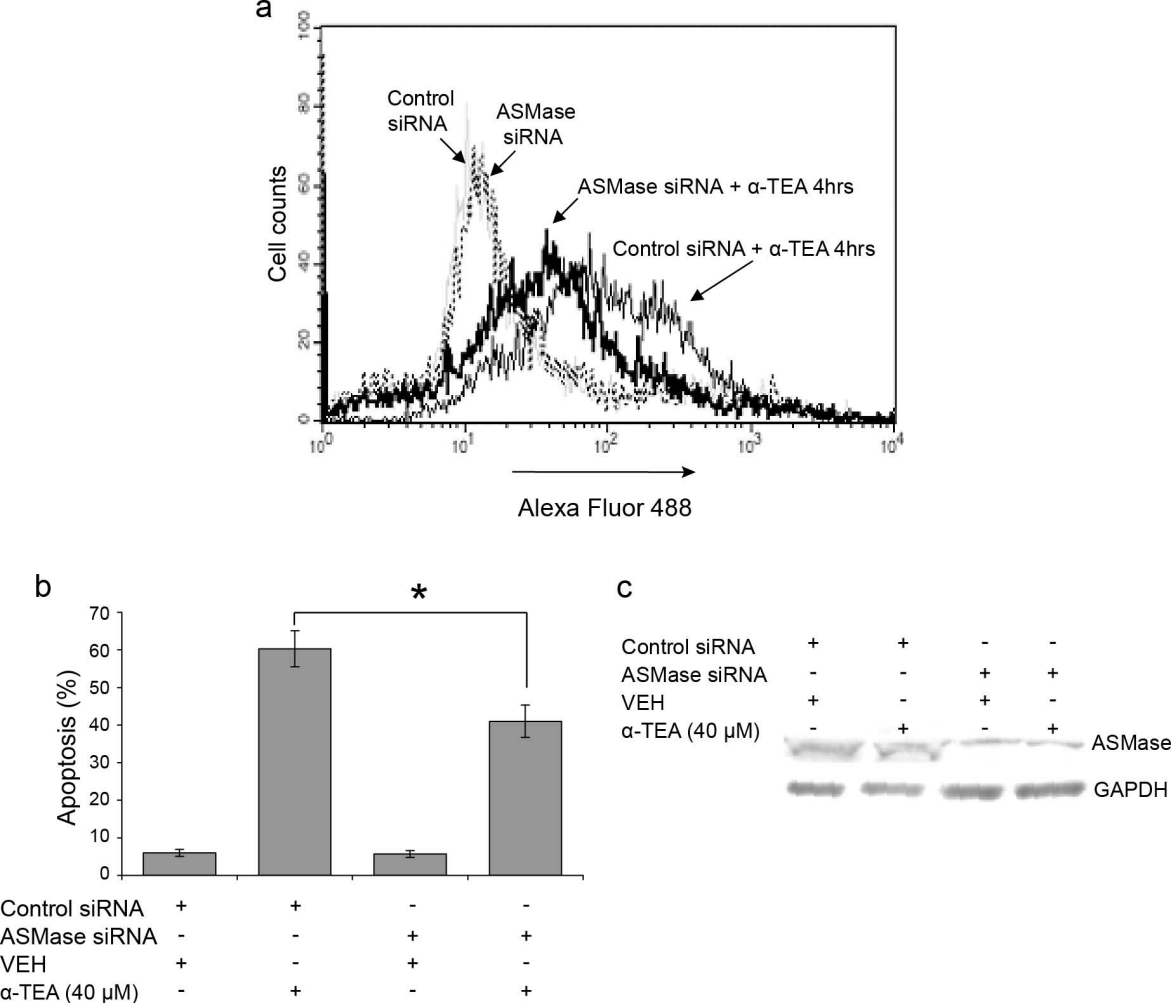
**siRNA to ASMase blocks α-TEA-induced apoptosis**. MDA-MB-231 cells were transfected with siRNAs to ASMase or control for 24 hrs followed by treating the cells with 40 μM α-TEA for 4 hrs. A, Ceramide levels in cellular membrane were detected by FACS analyses after immunofluorescent staining of living cells with primary ceramide antibody and fluorescence labeled secondary antibody. B, Annexin V/FACS analyses showed that siRNA to ASMase blocked α-TEA induced apoptosis. C, Western blot analyses was used to verified silencing efficiency of siRNA to ASMase using GAPDH as loading control. Data in A & C are representative of two independent experiments. Data in B are presented as means ± S.D. of three independent experiments. (* = significantly different, *P *< 0.05).

To further confirm the role of ASMase in α-TEA-induced apoptosis, siRNA to ASMase was used as an alternative method to knock down ASMase and to determine the effects on α-TEA-induced increases in cell surface ceramide and induction of apoptosis. siRNA to ASMase blocks α-TEA-induced increase in cell surface membrane ceramide detected by anti-ceramide antibody (15B4), further indicating that this antibody is specific for recognizing membrane ceramide (Figure 6A). In addition, siRNA to ASMase significantly reduced the ability of α-TEA to induce the cells to undergo apoptosis (Figure 6B). Western blot analyses show that the siRNA to ASMase reduced ASMase protein expression (Figure 6C), verifying silencing efficacy of siRNA to ASMase. These data further demonstrate that ASMase is involved in α-TEA-induced apoptosis and α-TEA-mediated increase in ceramide at the cell surface.

### ASMase is involved in α-TEA-induced accumulation of DR5, Fas and FADD in elevated ceramide-enriched membrane

**Figure 7:**
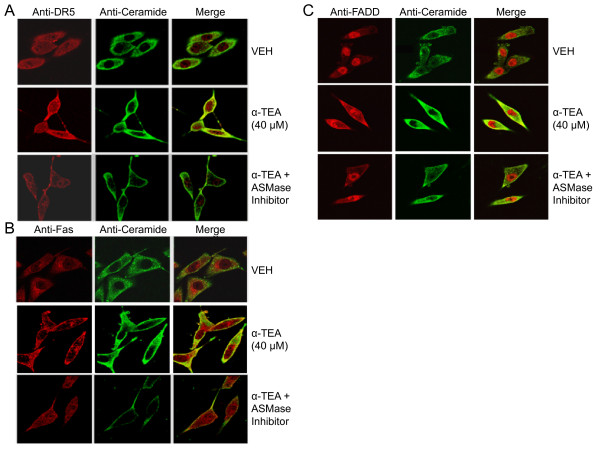
**ASMase is involved in α-TEA-induced accumulation of DR5, Fas, FADD and ceramide in cell surface membranes**. Cellular localization of DR5, Fas and FADD, as well as ceramide were detected by immunofluorescent staining of paraformaldehyde fixed cells with primary antibodies to DR5, Fas, FADD, and ceramide followed by fluorescent labeled secondary antibody. All data are representative of three independent experiments.

To see whether ASMase-mediated increase of cell surface ceramide is a contributor to α-TEA-induced accumulation of DR5, Fas and FADD in elevated ceramide-enriched membrane, the effects of ASMase inhibitor desipramine on α-TEA-induced co-localization of cell membrane death receptors Fas and DR5, as well as death adaptor protein FADD with ceramide were examined using immunofluorescent staining procedures (Figure 7A, B, &7C). As previously shown in Figure 3, Figure (7A, B and 7C) shows that α-TEA induces accumulation of DR5, Fas, and FADD in elevated ceramide-enriched membrane in comparison to VEH control (Figure 7A, B, &7C) and desipramine (ASMase inhibitor) reduces the ability of α-TEA-mediated events (Figure 7A, B, &7C). These data demonstrated that ASMase-mediated increase of ceramide in cell surface membrane is a contributor to α-TEA-induced accumulation of DR5, Fas and FADD in elevated ceramide-enriched membranes.

### Diet supplemented with α-TEA induced membrane ceramide accumulation in xenografted MDA-MB-231 cells

**Figure 8:**
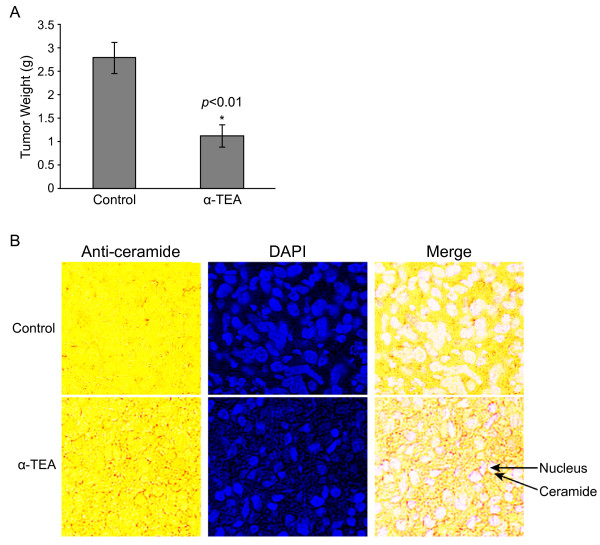
**α-TEA induced membrane ceramide accumulation in tumor tissue *in vivo***. A, Mean tumor weight ± S.D. for α-TEA treatment and control groups at euthanasia (N = 10). B, Tumor tissue sections (5 microns) from α-TEA treated and control were stained for ceramide by immunohistochemistry (brown) and with the DNA-fluorescent label DAPI (blue), and the two images were merged. Note: After merging the anti-ceramide stained tissue obtained from regular light with the blue nuclear staining obtained from UV light, the nuclear color turns to white in the merge image. Images are representative of three samples each of treatment and control group.

To determine if α-TEA induces increased levels of membrane ceramide *in vivo*, we examined membrane ceramide expression in 5 micron sections of tumor tissue taken from α-TEA or control treated immune compromised Nu/Nu mice bearing MDA-MB-231 xenografts. The tumor volume and tumor weight for α-TEA treatment group were significantly reduced in comparison to the control group after 24 days of treatment (*p *< 0.01) (Tumor volume data was published [27] and tumor weight data is shown in Figure 8A). It is important to note that no significant differences were observed in body weights between control and α-TEA treated groups. Previously published immunohistochemical analyses showed that TUNEL positive cells in the α-TEA treated group were significantly increased by 2.1 fold in comparison to control group [27]. Archived tumor samples were analyzed for ceramide expression by immunohistochemical analyses. Tumor tissue from α-TEA dietary supplemented animals exhibited increased levels of membrane ceramide (detected as brown stained circles around clear; i.e., white color of nuclei following merge) in comparison with absence of membrane-localized ceramide in control tumor tissue (Figure 8B). These data show that α-TEA increases membrane ceramide *in vivo*, suggesting that increased membrane ceramide plays a role in α-TEA antitumor actions *in vivo*.

## Discussion

α-TEA exhibits selective anticancer properties both *in vitro *and *in vivo *[10-24]. Mechanisms whereby α-TEA induces tumor cells to undergo apoptosis include activation of both death receptors (Fas and DR5) and JNK/p73/Noxa pro-apoptotic pathways; as well as suppression of anti-apoptotic mediators Akt, ERK, c-FLIP and survivin [15,16,18,20,21,28,29]. However, less is known about initiating events that lead to these desirable dual activities. New findings reported here include: 1) α-TEA triggers ASMase activation which involves translocation from cytosol to the cell surface membrane; 2) ASMase activation proceeds α-TEA-induced increase levels and aggregation of ceramide in the cell surface membrane; 3) α-TEA induces accumulation of DR5, Fas and FADD in elevated ceramide-enriched membrane; and 4) ASMase inhibitors (desipramine and siRNA to ASMase) decrease α-TEA-mediated membrane changes and apoptosis.

**Figure 9:**
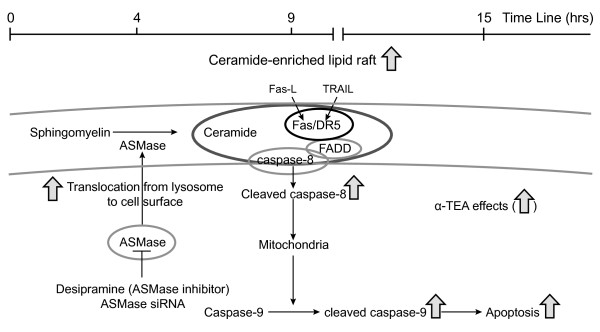
**Proposed model for α-TEA-induced ASMase/ceramide dependent apoptosis**. The model depicts α-TEA induction of ASMase translocation from cytosol (lysosomes) to the cell surface membrane and the generation of ceramide from hydrolysis of sphingomyelin as an early event (4 hrs) and the colocalization of DR5, Fas, and FADD with ceramide and induction of apoptosis as the cell fate (9-15 hrs). ASMase inhibitor desipramine or ASMase siRNA block α-TEA-initiated events.

Based on these data we propose the following sequence of events whereby α-TEA induces apoptosis in human MDA-MB-231 breast cancer cells (summarized in Figure 9). Within 30 min of α-TEA treatment ASMase activity starts to increase, and by 4 hrs there is evidence of ASMase translocation from the cytosol (intracellular compartments such as lysosomes) to the cell surface membrane. ASMase translocation is associated with increased levels of cell surface membrane ceramide. Co-localization of ceramide with Fas, DR5, and FADD and evidence of cleaved caspases 8 and 9 are observed by 9 and 15 hrs, while evidence of apoptosis (Annexin V expression and PARP cleavage) is observed at 15 and 24 hrs. Inhibition of ASMase activity using desipramine or ASMase expression using siRNA reduces the ability of α-TEA to increase cell surface membrane levels of ceramide, DR5, Fas and FADD and reduces α-TEA-induced apoptosis. In summary, α-TEA-induced apoptosis triggers an early ASMase-dependent generation of ceramide in the cell surface membrane of human MDA-MB-231 breast cancer cells, which is required for Fas and FADD relocation to the cell surface membrane and DR5, Fas and FADD co-localization in ceramide-enriched membrane domains.

The concept that vitamin E analogs can increase sphingomyelinase activity and ceramide in conjunction with apoptosis induction has been reported previously in other cancer cell types [30,31]. Weber *et al*. [30] showed that Jurkat human T lymphoma cells treated with the vitamin E analog, RRR-alpha-tocopheryl succinate (called vitamin E succinate), exhibited altered mitochondrial structure, generation of free radicals, and activation of the sphingomyelin cycle as early events in vitamin E succinate-initiated pro-apoptotic signaling. Based on a partial suppression of vitamin E succinate-induced apoptosis by the ASMase inhibitor desipramine, they demonstrated that activation of ASMase played a role in vitamin E succinate-induced apoptosis [30]. Our data are in agreement showing that ASMase activation is involved in apoptosis induced by another vitamin E derivative, α-TEA. Thus, activation of ASMase may be a common event in vitamin E analog-induced apoptosis that is not cell type specific. Likewise, our data are in agreement with the studies of Gu *et al*. [31] who showed that vitamin E succinate-induced apoptosis in human head and neck squamous cell carcinomas (HNSCC) was correlated with early increases in sphingomyelinase activity and ceramide levels. Furthermore, our data showing increased immunostaining of ceramide within cell surface membranes of tumor tissue derived from breast cancer bearing immune compromised mice fed α-TEA are in agreement with Gu *et al*.'s data showing a significant degree of immune staining of ceramide within the cellular membrane of HNSCC JHU-022 tumor tissue obtained from xenografted immune compromised mice injected i.p. with vitamin E succinate [31]. Thus, the ability of vitamin E analogs to induce elevated cell surface membrane levels of ceramide in cancer cells occurs both in cell culture and *in vivo *[31].

An important difference between the studies reported here and the studies cited above, is that we report a connection between early activation of ASMase and subsequent elevated cell surface membrane ceramide with co-localization with Fas, DR5 and FADD in membrane. Previously, we showed that α-TEA treatments increased Fas and Fas L mRNA and protein levels, as well as levels of cell surface membrane-associated Fas in human ovarian and prostate cancer cells and that functional knockdown of Fas and Fas-L attenuated α-TEA-induced apoptosis [20,21]. Recently, we showed that α-TEA treatment increases the expression of TRAIL and DR5 and that functional knockdown of DR5 and TRAIL blocked α-TEA's ability to induce apoptosis [15,16]. Thus, data suggest that α-TEA induction of apoptosis involves, at least in part, activation of extrinsic death receptor signaling. Information reported here suggests that α-TEA triggering of apoptosis via extrinsic death receptors involves upregulation of ceramide-enriched membrane domains via activation of ASMase.

Increases in cell surface membrane ceramide levels occur via sphingomyelinase catalyzed hydrolysis of sphingomyelin at the membrane. ASMase exists in two forms, a lysosomal and a secretory form [32]. Secretory ASMase translocates onto the outer leaflet of the plasma membrane from an intracellular location, presumably vesicular stores, to release ceramide within the raft-associated sphingomyelin pool, generating the ceramide required for raft clustering in response to various stress stimuli, including irradiation, death receptors signaling, and chemotherapeutic agents such as cisplatin and doxorubicin [32-38]. Therefore, ceramide in the cell surface membrane can function as an upstream or downstream mediator of extrinsic death receptor mediated apoptosis. Fas ligand and TRAIL have been shown to trigger accumulation of ceramide in membranes via recruitment of DISC and activation of caspase 8 [33,34,38], while another study showed that UV-induced accumulation of ceramide is FADD/caspase-8 independent [32]. Furthermore, ceramide-mediated formation of Fas- or DR5-clusters within the ceramide platform leads to triggering of death receptor-mediated apoptosis [4,9,33-35] which can be either ligand-dependent or -independent [32]. Data presented here show that increased levels and aggregation of membrane ceramide occur as early as 4 hrs after α-TEA treatment, while DR5/Fas/FADD membrane accumulation was not observed until 9 hrs after α-TEA treatment, suggesting that ceramide increase and aggregation may be an upstream event in α-TEA-induced Fas- and DR5-mediated apoptosis.

Several mechanisms have been proposed to be involved in the activation of ASMase, including ROS [33,39], lysosomal anionic lipids (bismonoacylglycerophosphate) and sphingolipid activator protein SAP-C [40]. Recent data show that PKCδ contributes to the translocation and activation of ASMase via phosphorylation of ASMase at ser-508 by PMA [37]. How α-TEA regulates ASMase activation is not known. Our preliminary data do not support the role of ROS in α-TEA-induced ASMase activation since ROS was not induced by α-TEA and the antioxidant NAC did not block α-TEA-induced increases in the levels of membrane ceramide (data not shown). Since α-TEA is a detergent-like lipid it is possible that α-TEA targets lysosomes initiating activation of ASMase. This remains to be determined.

Ceramide action is determined within the context of other stimuli and by the subcellular location of its production. Beside ASMase hydrolysis of sphingomyelin to mediate ceramide levels at the cell surface, there are two more pathways in which intracellular ceramide can be generated, namely, the de novo synthesis pathway (located in the endoplasmic reticulum) and the salvage pathway (located in late endosomes/lysosomes) [41]. Both of these pathways generate ceramide and others metabolites, which may contribute to apoptosis induction via mechanisms that differ from ceramide's role at the cell surface. For example; dihydroceramide, an intermediate in the de novo synthesis pathway, has been reported to trigger apoptosis and is upregulated by γ-tocopherol [42]. Glucosylceramide, an intermediate in the salvage pathway, shows anti-apoptotic properties and can be converted to ceramide by cerebrosidase (glucohydrolases) [43]. Whether α-TEA regulates these pathways is not known and beyond the scope of this studies; however, we do know that de novo synthesis pathway is not involved in α-TEA-induced apoptosis based on the fact that the de novo ceramide synthesis inhibitors myriocin and fumonisin B1 did not block α-TEA-induced apoptosis (data not shown). These inhibitor studies also suggest that ceramide synthase-mediated ceramide may not be involved in α-TEA-induced apoptosis since fumonisin B1, an inhibitor of ceramide synthase, did not block α-TEA- induced apoptosis.

To study how α-TEA regulates ceramide levels in cellular membranes we used anti-ceramide antibody (15B4), which provides special benefit to study ceramide in its cellular context, such as subcellular localization of ceramide. Although this antibody has been used in many studies, the specificity of this antibody is still questioned. For example, lipid overlay assays show that the 15B4 anti-ceramide antibody recognizes not only C16- and C24-ceramide, but also other lipids such as dihydroceramide, sphingomyelin and phosphatidylcholine [44]. However, it is highly specific for ceramide and does not cross-react with sphingomyelin, cholesterol or other phospholipids under more physiologically relevant *in vitro *and *in vivo *conditions [34,44]. In the studies reported here, both ASMase inhibitor desipramine and siRNA to ASMase blocked α-TEA-induced increases in 15B4 antibody-recognized phospholipids in membrane clearly indicating that anti-ceramide (15B4) is specific for recognizing membrane ceramide and confirming that α-TEA effects are dependent on ASMase-mediated ceramide increases in membrane.

## Conclusion

In summary, our findings demonstrate that activation of ASMase, leading to increases in cell surface membrane ceramide, is a critical early event for extrinsic death receptor signaling in α-TEA-induced apoptosis. This is the first study that identifies possible initiation event in α-TEA-induced apoptosis.

## Abbreviations

ASMase: acid sphingomyelinase; α-TEA: Alpha-tocopherol ether-linked acetic acid analog; DISC: death inducing signaling complex; DR5: Death receptor 5; FADD: Fas-Associated protein with Death Domain; TRAIL: TNF related apoptosis induced ligand.

## Competing interests

US and international patents on α-TEA are held by the Research Development Foundation. Kimberly Kline and Bob G. Sanders are listed as inventors. No commercial applications or financial gain have been realized.

## Authors' contributions

JL carried out immuno-fluorescent assays, ceramide detection, ASmase detection assay, Annexin V staining and drafted the manuscript. WY conceived the study, participated in the design, performed the statistical analysis, carried out cell culture and drafted/revised the manuscript. RT carried out western blot assays. SKP provided technical help with detection of ceramide expression and apoptosis assay. AX helped in cell culture. BGS and KK conceived the study, drafted/revised the manuscript and provided critical analysis. All authors read and approved the final manuscript.
